# Hemodynamic and microcirculatory early adaptations following transcatheter aortic valve implantation (TAVI): A physiological pilot study

**DOI:** 10.1111/eci.70156

**Published:** 2025-12-02

**Authors:** Stanislas Abrard, Sarah Mauler, Ivo Neto Silva, Dyonisios Adamopoulos, Stéphane Bar, Andres Hagerman, Raoul Schorer, Bernardo Bollen Pinto, Georgios Rovas, Nikolaos Stergiopulos, Christoph Ellenberger, Stéphane Noble, Karim Bendjelid

**Affiliations:** ^1^ Geneva University Hospitals, and Hemodynamic Research Lab, Geneva Medical School Geneva Switzerland; ^2^ Department of Anesthesiology University Hospitals of Geneva Geneva Switzerland; ^3^ Department of Anesthesiology and Critical Care Medicine Hospices Civils de Lyon, Edouard Herriot Hospital Lyon France; ^4^ Research on Health‐Care Performance (RESHAPE) INSERM U1290, University Claude Bernard Lyon 1 Lyon France; ^5^ Faculté de Médecine Lyon Est Université Claude Bernard Lyon 1 Lyon France; ^6^ Cardiology Division, Department of Medicine Geneva University Hospitals Geneva Switzerland; ^7^ Adult Intensive Care Division Hôpitaux Universitaires de Genève Geneva Switzerland; ^8^ Department of Anesthesiology and Critical Care Medicine Amiens University Medical Centre Amiens France; ^9^ Swiss Federal Institut of Technology (EPFL), Laboratory of Hemodynamics and Cardiovascular Technology (LHTC) Lausanne Switzerland

**Keywords:** endothelial function, hemodynamics, microcirculation, tissue perfusion, Transcatheter aortic valve implantation, vascular adaptation

## Abstract

**Background:**

Transcatheter aortic valve implantation (TAVI) abruptly relieves aortic stenosis. The consequences for the peripheral vascular network, organ perfusion and postoperative organ dysfunction remain unclear. This study assessed hemodynamic and microcirculatory changes after TAVI, and their association with postoperative organ dysfunction.

**Methods:**

This prospective, single‐center physiological study included 20 patients with severe aortic stenosis undergoing transfemoral TAVI at Geneva University Hospitals (January–June 2024). Hemodynamic and microcirculatory assessment included arterial stiffness (tonometry), temperature gradients (T grad), reactive hyperemia (near‐infrared spectroscopy and photoplethysmography) and plasma vascular endothelium growth factor (VEGF) concentrations before and after TAVI. The primary outcome was perioperative changes in macro‐ and microcirculatory parameters; secondary outcomes were organ dysfunction within 7 days.

**Results:**

TAVI immediately increased aortic pressures and amplified pressure waves. By day 1, central‐peripheral T grad decreased, perfusion index rose (from 2.5 [0.9–4.2] to 3.9 [1.9–5.5]; *p* < 0.05), and tissue oxygen re‐saturation slope increased (from 2.6 [1.5–3.4] to 3.9 [2.8–4.7] %/s; *p* < 0.05), independent of macrocirculatory parameters. Large artery stiffness decreased, despite a reduction in the total arterial compliance, without changes in small‐vessel resistance. Cardiac index changes showed wide interindividual variability and correlated with vascular and VEGF dynamics. Patients with postoperative organ dysfunction had higher baseline VEGF (52.9 vs. 28.7 pg/mL, *p* = 0.033) and greater postoperative increases.

**Conclusion:**

TAVI induces rapid macro‐ and microcirculatory changes, with early tissue perfusion improvement despite transient microcirculatory reserve impairment. VEGF dynamics were associated with postoperative complications, suggesting endothelial activation as a marker of vulnerability and linking baseline endothelial status to vascular adaptation and outcomes.

## Graphical abstract

This pilot physiological study, involving 20 patients with severe aortic stenosis, shows that transcatheter aortic valve implantation (TAVI) induces rapid changes in both macrocirculatory parameters, reflecting the release of chronic obstruction. We observed improved tissue perfusion by postoperative Day 1, independent of macrocirculatory parameters. Cardiac index changes correlated with vascular and VEGF dynamics. Patients with postoperative organ dysfunction had higher baseline VEGF and greater postoperative increases. These findings highlight the role of microcirculation in recovery after TAVI and suggest potential prognostic markers for patient stratification.

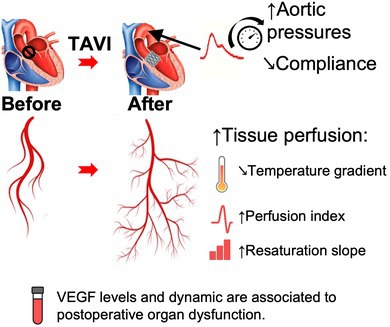

## 1 INTRODUCTION

Severe aortic valve stenosis (AS) which affects 2%–9% of individuals aged over 65, significantly alters cardiovascular hemodynamics,^1^ by increasing left ventricular afterload and impeding systemic circulation outflow. The valvular obstruction finally leads to chronic pressure overload and ventricular remodelling, particularly hypertrophy. Pathological neovascularization and chronic inflammation significantly impact AS progression and outcomes following TAVI.^2^, ^3^ The remodelling progressively reduces ventricular filling and cardiac output, and causes heart failure.^4^ Without timely valve replacement, symptomatic severe AS is associated with a high mortality.^5^ Transcatheter Aortic Valve Implantation (TAVI) provides a sudden reduction in ventricular afterload, abruptly changing pressure regimes within the cardiovascular system. While this intervention is now the standard of care in frail, elderly and multimorbid patients.^6^, ^7^ The pathophysiology of the vascular adaptation remains insufficiently understood. Elderly and frail patients often have diminished adaptability, risking maladaptive responses and organ dysfunction. Preliminary research suggests that microcirculatory biomarkers such as baseline vascular endothelial growth factor (VEGF) and arterial stiffness may predict postoperative hemodynamic improvement and recovery but remain insufficiently explored.^8^, ^9^, ^10^, ^11^, ^12^, ^13^

Preoperative risk assessment in this setting remains challenging. Established cardiac risk models like EuroSCORE II and STS inadequately capture the profile of TAVI patients, typically older, frailer and with multiple comorbidities.^14^, ^15^ Increasingly, impaired microcirculation and chronic inflammation are recognized as influential factors for postoperative outcomes,^2^, ^3^, ^16^, ^17^ including organ dysfunction yet they remain unaddressed in TAVI risk assessments.^8^, ^9^ In this context, a physiological approach may provide valuable insights. Understanding vascular and microcirculatory early adaptation to TAVI could improve our comprehension of postoperative trajectories and help explain the risk of organ dysfunction.

The present pilot observational pathophysiological study aims to prospectively assess microcirculatory and hemodynamic changes before and after TAVI, correlating VEGF levels with clinical outcomes.

## 2 METHODS

### 2.1 Study design and setting

This is a prospective, single‐center, investigator‐initiated, observational, physiological, pilot study conducted at Geneva University Hospitals (HUG) between January and June 2024. Patients with severe AS scheduled for TAVI were recruited during preoperative consultation and followed for 30 days. The study was approved by the local Ethics Committee (25th October 2023, Protocol 2023‐01118) and registered at ClinicalTrials.gov (NCT06154642). Written informed consent was obtained from all participants.

### 2.2 Participants

Eligible patients were ≥18 years with severe AS scheduled for transfemoral TAVI. Main exclusion criteria included non‐femoral access, planned general anaesthesia, left ventricular ejection fraction <40%, advanced comorbidities or known cognitive impairment, unable to provide consent. The details of the inclusion and exclusion criteria are presented in Supplementary Material.

### 2.3 Follow‐up and data collection

**FIGURE 1:**
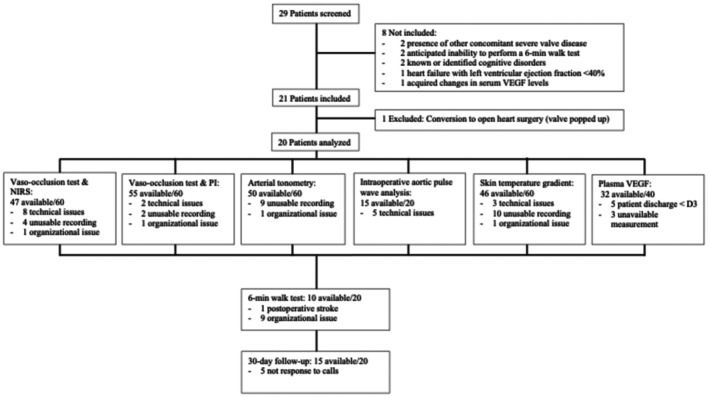
Study flow chart. NIRS, Near infrared spectrometry; PI, Perfusion index; VEGF, vascular endothelium growth factor.

Assessments were performed at baseline in cath‐lab just before TAVI, intraoperatively, and at 3 h (H + 3), Day 1 (D + 1) and Day 3 (D + 3) post‐procedure, with clinical follow‐up at Day 7 (D + 7) and Day 30 (D + 30) (Supplement Material– Figure 1). Data included hemodynamic and microcirculatory measurements (arterial stiffness, cutaneous temperature gradients (T grad), reactive hyperemia, perfusion index, tissue oxygen saturation (StO_2_) and plasma VEGF concentrations) as well as transthoracic echocardiographic (TTE) and laboratory parameters.

Microcirculatory parameters were measured on resting participants instructed not to move or speak during the procedure. Arterial stiffness of large and small vessels was measured noninvasively using applanation tonometry with a pressure sensor placed on the radial artery to assess pulse wave characteristics (HDI Pulsewave CR‐2000, Eagan, MN, USA). The T grad was measured using surface thermometers (Biopac Systems Inc. CA, USA) placed on the forearm, midway between the wrist and the elbow and the fingertip opposite to the nailbed, with the gradient calculated as the difference in skin temperature between the two sites. Reactive hyperemia was assessed via a vaso‐occlusion test (VOT) using a pneumatic cuff inflated (50 mmHg up arterial pressure) for 3 min followed by measurement of reperfusion using photoplethysmography on the index finger (Masimo Radical 7, Masimo Corp, CA, USA) and StO_2_ by near‐infrared spectroscopy (NIRS) (INVOS 5100C, Medtronic, CO, USA). Collected parameters included perfusion index peak amplitude, time to peak from photoplethysmography, as well as tissue resaturation rate (rStO_2_) from NIRS. Plasma VEGF concentrations were measured from venous blood samples taken via existing vascular access using enzyme‐linked immunosorbent assay (ELISA) methodology (R&D Systems Inc., MN, USA) and analyzed at the hospital laboratory. The intra‐aortic pressure signal was recorded using standard cath‐lab monitoring systems (Biopac Systems Inc. CA, USA) and stored for offline analysis as described by Pagoulatou et al.^11^ The complete TTE in a supine position was performed before the procedure and between D + 1 and D + 2 post‐TAVI in all study participants by an experienced cardiologist. We collected data on left ventricular geometry and proximal velocity profile, which were acquired in the left ventricular outflow tract via Pulsed Wave Doppler in the standard apical 5‐chamber view. The aortic flow waveform was subsequently derived after calibration for the invasively measured systolic volume. Aortic valve assessment and qualitative evaluation of other valve abnormalities (mitral, tricuspid) were extracted from the standard echocardiographic reports.

### 2.4 Outcomes

The primary outcome was the change in microcirculatory parameters after TAVI. Secondary outcomes included postoperative organ dysfunction within 7 days (composite of AKI, cardiovascular failure or neurocognitive disorder). Additional outcomes included functional capacity on Day 6, major adverse cardiovascular/kidney events within 30 days, and all‐cause mortality. Functional capacity was evaluated using the 6‐min walk test (6MWT), which was performed by trained staff.

Additional methodological details (including measurement techniques, sedation protocol and bias control procedures) are provided in Supplement Material–Detailed protocol.

### 2.5 Statistical methods

Continuous variables were expressed as medians [IQR] and categorical variables as counts (%). Within‐patient changes were analyzed with the Wilcoxon signed‐rank test; between‐group comparisons were performed using the Mann–Whitney *U* test or Fisher's exact test. No formal sensitivity analyses or adjustments for multiple comparisons were planned due to the study's exploratory nature and small sample size. The planned sample size of this physiological pilot study of 20 patients was determined according to our institution's annual TAVI procedure volume (80–90 cases), ensuring the feasibility of recruitment within a 12‐month period. Analyses were performed with SPSS software version 23 (IBM Corp., USA); *p*‐value <0.05 was considered significant. Missing data were imputed only in linear models; otherwise, analyses used available data.

## 3 RESULTS

### 3.1 Study participants

During the 6‐month inclusion period, 29 patients scheduled for transfemoral TAVI at HUG were screened; 21 met the inclusion criteria, but one was excluded due to intraoperative conversion to open surgery, and 20 patients were analysed (Figure 1). Table 1 presents patient characteristics. Median age was 84 [78–87] years, 95% had Grade 4 AS, and 60% had Stage 2–3 cardiac damage. Follow‐up to Day 7 was complete for all patients, and the 6MWT was available in 10/20 patients (Figure 1). Intraoperative events were described (Table 2).

**TABLE 1:** Characteristics of patients.

Characteristic	Total cohort
*N*	20 (100.0)
Male sex	10 (50)
Age at the enrollment, years	84 [78–87]
Body mass index, kg/m^2^	24.9 [22.4–26.8]
Medical conditions	
Diabetes mellitus	5 (25)
Hypertension	16 (80)
Chronic kidney disease	6 (30)
Peripheral artery disease	2 (10)
Stroke	3 (15)
Chronic respiratory disease	
Obstructive apnea	2 (10)
Mild COPB	1 (5)
*Cardiopathy*	
Aortic stenosis	
Mean gradient (mmHg)	48 [37–60]
Surface (cm^2^)	0.77 [0.66–0.84]
Vmax (m/s)	4.49 [4.07–4.76]
Valve calcic score	2775 [1878–4091]
Valvular grading severity	
Grade 3	1 (5)
Grade 4	19 (95)
Angina	2 (10)
Dyspnea	16 (80)
Syncope	2 (10)
Associated valvulopathy	
Mild aortic insufficiency	11 (55)
Mild/Moderate mitral insufficiency	14 (70)
Mild/Moderate tricuspid insufficiency	13 (65)
Cardiopathy (other than valvular)	12 (60)
Atrial fibrillation/flutter	8 (40)
Myocardial infarction	8 (40)
Stenting	7 (35)
Coronary artery bypass grafting	2 (10)
Left ventricle ejection fraction, %	60 [60–65]
Cardiac damage staging	
Stage 1	8 (40)
Stage 2	4 (20)
Stage 3	8 (40)
EuroSCORE II	7 [5–9]
STS Morbi‐mortality	0.1040 [0.0794–0.1438]
STS AKI	0.0146 [0.0099–0.0349]
STS‐TAVR score	0.0251 [0.0183–0.0368]
Preoperative biology	
Creatinine, μmol/L	80 [68–113]
NTproBNP, pg/mL	1230 [228–2397]
Platelet count, G/L	255 [214–317]
Hemoglobinemia, g/L	124 [116–136]
Leukocytes, G/L	6.9 [5.6–8.6]
CRP, mg/L^a^	2.1 [0.8–6.1]
VEGF, pg/mL	36.3 [28.7–53.6]
Preoperative medications	
Beta‐blocker	8 (10)
ACE inhibitors or ARB	7 (35)
Antiplatelet therapy	7 (35)
Anticoagulant	8 (40)
Oral antidiabetic therapy	7 (35)
Psychotropic therapy	7 (35)
Calcium channel blocker	7 (35)
Statins therapy	12 (60)
Proton pump inhibitor	5 (25)

*Note*: Data are expressed as median [interquartile range] or number (percentage of the entire cohort).

Abbreviations: ACE inhibitor, angiotensin converting enzyme inhibitor; ARB, angiotensin II receptor blocker; CABG, coronary arterial bypass grafting; CPB, cardiopulmonary bypass; CRP, C reactive protein; SOFA, sequential organ failure assessment.

^a^ Limit of detection <4 mg/L.

**TABLE 2:** Intra‐ and postoperative outcomes.

	Total cohort
Vasopressors, %	13 (65)
Before valve implantation, *n* (%), median dose μg/kg/min	13 (65), 0.041 [0.027–0.050]
Norepinephrine	11 (55)
Phenylephrine	2 (10)
After valve implantation, *n* (%) median dose μg/kg/min	6 (30), 0.029 [0.009–0.046]
Norepinephrine	5 (25)
Phenylephrine	1 (5)
Intra‐operative complication	
General anaesthesia for agitation	2 (10)
Third‐degree atrioventricular block	3 (15)
Femoral arterial dissection	1 (5)
Postoperative organ dysfunction within 7 days, *n* (%)	11 (55)
Confusion at Day 1, *n* (%)	4 (20)
Postoperative vasopressor >2 h, *n* (%)	6 (30)
Postoperative AKI, *n* (%)	6 (20)
Postoperative vasopressors doses	
H + 3, *n* (%), median dose μg/kg/min	2 (10), 0.042 [0.024‐]
D + 1, *n* (%), median dose μg/kg/min	0
Postoperative biological course	
VEGF evolution at D + 3, pg/mL	+49.8 [−4.8–69.4]
Postoperative peak of troponins, ng/L	186 [114–255]
Kidney	
AKI KDIGO 1, *n* (%)	5 (25)
Postoperative peak of creatinine (peak‐baseline), μmol/L	3 [−2,16]
Stroke, *n* (%)	1 (5)
6 min walk test, m	321 [164–369]
% of predicted distance	77 [47–91]
Length of follow‐up, days	31 [18–32]

### 3.2 Primary outcomes

**FIGURE 2:**
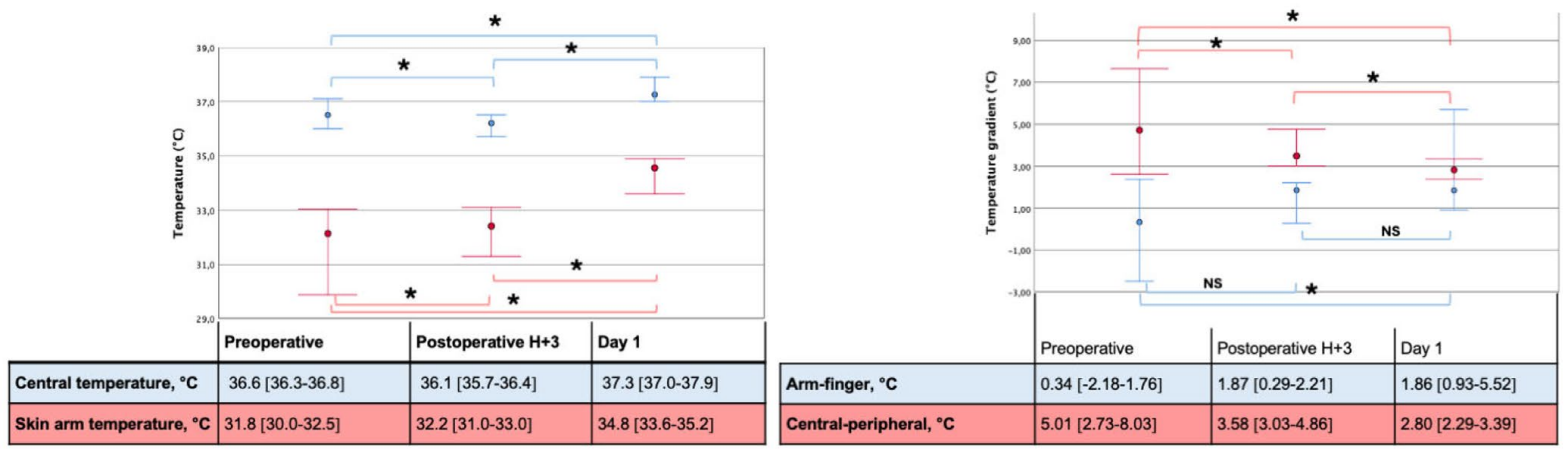
Temperature measurements. Temperatures (left; red = skin arm; blue = central) and temperature gradients (right; red = central‐peripheral temperature gradient; blue = arm‐finger temperature gradient) are presented at three different timepoints. Measurements were compared by the Wilcoxon test. *p < 0.05.

Key perioperative trends are shown in Tables 3 and 4, Figure 2. The arm‐finger T grad, which was minimal preoperatively and negative for some patients, increased significantly at D + 1 (*p* = 0.023); central‐peripheral T grad decreased significantly at H + 3 (*p* = 0.046) and D + 1 (*p* = 0.013). Baseline perfusion index tended to increase from 2.5 [0.9–4.2] to 3.6 [2.4–5.9] at H + 3 (*p* = 0.098) and, significantly, 3.9 [1.9–5.5] at D + 1 (*p* = 0.011); while the reactive hyperemia and time to peak perfusion index tended to be lower and delayed at H + 3 (68 [16–258] to 35 [16–61] %, *p* = 0.234; 43 [22–61] to 60 [45–71] s, *p* = 0.070, respectively) but normalized at D + 1 (Table 3, Figure S2). StO_2_ remained stable postoperatively; rStO_2_ tended to decrease at H + 3 (*p* = 0.163), then significantly increased at D + 1 (*p* = 0.012), indicating enhanced microvascular reactivity. Median VEGF levels increased by +95% at D + 3 (*p* = 0.033) with high inter‐individual variability (Table 2, Figure S3).

**TABLE 3:** Reactive hyperemia parameters after vaso‐occlusion test.

Perfusion parameters	Preoperative	Postoperative H + 3	Day 1
PI baseline	2.5 [0.9–4.2]*	3.6 [2.4–5.9]	3.9 [1.9–5.5]
Ratio PI peak, %	68 [16–258]	35 [16–61]	50 [26–104]
Time to PI peak, s	43 [22–61]	60 [45–71]	48 [32–66]
StO_2_ baseline, %	53 [46–61]	53 [46–55]	46 [39–67]
Peak StO_2_ amplitude, %	24 [16–29]	25 [11–30]	20 [13–25]
rStO_2_, %/s	3.3 [2.7–4.2]	2.6 [1.5–3.4]*	3.9 [2.8–4.7]

*Note*: Measurements were compared by the Wilcoxon test.

Abbreviations: PI, perfusion index; rStO_2_, recovery slope of tissular oxygen saturation; rStO_2_, tissular oxygen saturation.

* *p* < 0.05 compared to Day 1.

**TABLE 4:** Evolution of hemodynamic and microcirculatory parameters.

	Preoperative	Postoperative H + 3	Day 1	Pre vs. H3	Pre vs. D1	H3 vs. D1
Macrohemodynamic						
Systolic arterial pressure, mmHg	147 [130–157]	120 [107–134]	136 [120–146]	**0.003**	0.079	**0.018**
Mean arterial pressure, mmHg	101 [90–112]	82 [70–97]	91 [83–99]	**<0.001**	**0.008**	0.121
Diastolic arterial pressure, mmHg	70 [54–85]	60 [48–73]	63 [57–69]	**0.027**	0.073	0.235
Pulse rate, bpm	70 [55–85]	65 [50–70]	74 [67–83]	0.064	0.156	**<0.001**
TTE						
TTE VTI, cm	20.9 [18.4–23.6]	‐	19.0 [17.0–26.9]	‐	0.616	‐
Cardiac index, L/min/m^2^	2.74 [2.57–3.18]		2.56 [2.38–3.31]			
Pulse tonometry						
Cardiac index, L/min/m^2^	2.41 [2.24–2.55]	1.99 [1.74–2.36]	1.84 [1.66–1.93]	**0.016**	**0.001**	**0.016**
Systemic vascular resistances, dyne.s.cm^−5^	1878 [1660–2291]	1877 [1582–2497]	2164 [1974–2505]	0.379	**0.030**	**0.007**
Total vascular impedance, dyne.s.cm^−5^	233 [189–370]	255 [192–346]	299 [258–353]	0.865	**0.002**	**0.030**
Stiffness of large‐calibre arteries, mL/mmHg	9.5 [4.9–11.3]	7.6 [5.2–11.5]	5.9 [5.2–6.4]	0.802	**0.004**	**0.006**
Stiffness of small‐calibre arteries, mL/mmHg	2.9 [2.0–4.2]	2.6 [1.5–3.9]	2.9 [1.9–4.1]	0.396	0.570	0.366

*Note*: Measurements were compared by the Wilcoxon test. Bold: *p* < 0.05.

Abbreviation: TTE VTI, transthoracic echocardiography measure of subaortic velocity–time integral.

Echocardiographic stroke volume (VTI) showed no significant evolution at D + 1 (*p* = 0.616), whereas cardiac index (CI) measured by tonometry showed a strong decrease (*p* = 0.001). Intra‐aortic pulse wave analysis (Table 5) showed that CI, heart rate, pulse wave velocity and reflection coefficient remained stable. The main changes concerned an increase in systolic, pulse, maximal slope of aortic pressures and time reduction to reach the peak of aortic systolic pressure; as well as a decrease in wave transit time. Wave separation analysis showed an increase in forward wave pressure amplitude, its maximal slope, forward wave flow and an earlier forward wave pressure peak occurred after TAVI. Backward wave amplitude also increased, with an earlier peak and steeper slope. The two‐element Windkessel model showed stable total vascular resistance, while total arterial compliance decreased significantly post‐TAVI with regard to new pressure conditions.

**TABLE 5:** Effect of TAVI on vascular parameters assessed via the pulse pressure method and on the aortic pressure wave components assessed via frequency‐based wave separation analysis.

	Pre‐TAVI	Post‐TAVI	*p* value
Aortic flow			
Cardiac index, L/min/m^2^	2.54 [2.00–3.31]	2.41 [2.22–3.73]	0.233
Heart rate, bpm	67 [55–77]	67 [60–79]	0.060
Pulse wave velocity, m/s	5.76 [4.63–7.82]	5.29 [5.02–6.20]	0.460
Aortic pressure			
Aortic SBP, mmHg	126 [117–141]	149 [136–159]	0.006
Aortic DBP, mmHg	57 [46–68]	57 [44–70]	0.753
Aortic MBP, mmHg	84 [75–92]	87 [79–99]	0.064
Aortic PP, mmHg	72 [49–96]	97 [72–107]	0.004
Time to aortic SBP, s	0.30 [0.26–0.33]	0.24 [0.21–0.26]	0.001
Maximal aortic pressure slope, mmHg/s	397 [290–475]	668 [589–857]	0.001
Aortic inflection point, mmHg	60 [52–71]	61 [52–74]	0.489
Time to aortic inflection point, s	0.04 [0.03–0.08]	0.04 [0.03–0.04]	0.099
Aortic augmentation pressure, mmHg	69 [42–87]	91 [66–102]	0.003
Aortic augmentation index, %	0.95 [0.90–0.97]	0.95 [0.93–0.96]	0.886
Wave separation analysis			
Characteristic impedance, dyne.s.cm^−5^	185 [139–217]	183 [148–200]	0.394
Forward wave amplitude, mmHg	52 [45–62]	71 [52–78]	0.001
Time to forward wave peak, s	0.28 [0.26–0.29]	0.21 [0.17–0.23]	0.001
Maximal forward wave slope, mmHg/s	499 [412–582]	659 [586–811]	0.005
Forward wave flow, mL/s	371 [335–435]	517 [446–633]	0.005
Backward wave amplitude, mmHg	24 [14–37]	38 [29–42]	0.016
Time to backward wave peak, s	0.37 [0.36–0.44]	0.29 [0.26–0.34]	0.001
Maximal backward wave slope, mmHg/s	203 [140–252]	310 [273–460]	0.003
Wave transit time, m/s	0.13 [0.12–0.35]	0.09 [0.07–0.16]	0.028
Reflection coefficient	0.68 [0.55–0.77]	0.63 [0.54–0.70]	0.925
Backward wave flow, mL/s	172 [106–255]	292 [203–345]	0.053
Backward/forward wave flow ratio	0.40 [0.33–0.70]	0.53 [0.45–0.62]	0.593
2‐Element Windkessel parameters			
Total vascular resistance, dyne.s.cm^−5^	1273 [1147–2068]	1460 [1121–1614]	1.000
Total arterial compliance, mL/mmHg	0.56 [0.42–0.81]	0.45 [0.38–0.65]	0.048

*Note*: *N* = 15. S/D/MBP: Systolic/Diastolic/Mean blood pressure. Comparisons between groups were made using the Wilcoxon test.

## 4 SECONDARY OUTCOMES

Postoperative complications included cognitive disturbance (20%), need for vasopressor support >2 h (30%) and AKI (30% mostly KDIGO stage 1) that are associated with different changes in baseline StO_2_ and peak StO_2_ amplitude (Table 2, Table S1).

## 5 EXPLANATORY ANALYSIS

CI changes varied inter‐individually and correlated with post‐TAVI vascular compliance (*r* = 0.726; *p* = 0.002) and large artery stiffness (*r* = 0.805; *p* = 0.002) (Figures S4 and S5). Baseline VEGF was negatively correlated with reactive hyperemia indices and temperature gradients (Figure S6). Microcirculatory indices did not show any correlation with the CI or macrocirculation (Figure S7). Changes in plasma VEGF levels at D + 3 correlated with changes in StO_2_ (*r* = 0.681; *p* = 0.044) and were negatively correlated with changes in CI (*r* = −0.806; *p* = 0.009) (Figure S8), post‐TAVI vascular compliance (*r* = −0.705; *p* = 0.034), and large artery stiffness (*r* = −0.665; *p* = 0.026).

In the upper tercile of CI change (>0.5 L/min/m^2^), composed exclusively of women, baseline measures showed lower central‐peripheral and arm‐finger T grads, as well as a reduced and delayed reactive hyperemia (Table S2). Unlike other patients, they exhibited no postoperative rise in VEGF nor decrease in large‐artery stiffness and central‐peripheral T grad.

## 6 DISCUSSION

The present prospective physiological pilot study aimed to characterize the early evolution of microcirculation and hemodynamics before and after TAVI. The findings support the hypothesis that TAVI induces rapid and measurable changes in both macro‐ and microcirculation. Our main findings are that (1) Significant postoperative improvements in tissue perfusion were not correlated to CI or macrocirculatory improvement. (2) Immediate increases in aortic systolic pressure, pulse pressure, and forward/backward wave amplitude after valve deployment reflected the removal of post‐ventricular obstruction. (3) Large‐calibre arterial stiffness and arterial compliance significantly decreased post‐TAVI without changes in small‐calibre artery stiffness and vascular resistances. (4) Gender‐associated interindividual variability in CI change, correlated with vascular compliance, artery stiffness and VEGF level dynamics. (5) Higher baseline VEGF level was associated with postoperative organ dysfunction and both macro‐ and microcirculatory parameters.

TAVI produces a sudden and profound change in left ventricular afterload by relieving the fixed obstruction caused by aortic stenosis. When the obstruction is lifted, an increase in the strain of the left ventricular ejection volume (pulse wave) on the existing vascular tree is observed. However, despite increased pressure transmission, systolic ejection volume did not rise, likely due to the limited vasodilatory capacity of the vascular tree after years of AS adaptation, as reflected by stable resistance and reduced compliance. In a similar study on vascular adaptation in 33 AS cases, Pagoulatou et al.^11^ showed that TAVI resulted in an immediate increase in aortic systolic pressure without changes in vascular resistance, with a more pronounced forward wave.

In our study, CI measurements obtained by tonometry were inconsistent with those from TTE and intra‐aortic pulse wave analysis, which were robust and concordant. This discrepancy is likely explained by the mathematical coupling in the tonometry algorithm. The device estimates CI using a simple linear formula based on ejection duration, an approach that has shown only modest accuracy in healthy subjects. After TAVI, this method becomes particularly unreliable because (1) prosthetic valves shorten ejection duration, (2) they generate steeper pressure gradients and (3) valve area no longer correlates with body surface area, one of the original assumptions of the formula.^18^ In our cohort, the 20% reduction in ejection duration after TAVI paralleled almost exactly the 16% fall in CI estimated by tonometry, illustrating this artifact. By contrast, pulse wave analysis derived from intra‐aortic catheter measurements does not rely on these assumptions and is methodologically superior, as reflected in the more consistent results we observed. These considerations should be acknowledged as a limitation and help explain the conflicting findings.

Microcirculatory data showed transient impairment in functional microcirculatory reserve at H + 3 (reduced reactive hyperemia, trend to delayed PI peak) but preserved StO_2_, indicating a compensatory mechanism to maintain tissue perfusion (Table 3). At D + 1, reactive hyperemia and rStO_2_ improved, reflecting the recovery of functional microcirculatory reserve. The lack of correlation between the CI and microcirculatory parameters indicates a situation of decoupling between the two circulations.^19^ After TAVI, central‐peripheral T grad decreased, suggesting improved tissue perfusion through better mixing of thermal compartments.^20^ This benefit was mainly proximal, with arm temperature rising while finger temperature remained stable, increasing the arm‐finger T grad. Some patients had a negative preoperative arm‐finger T grad due to chronic distal vascular adaptation to aortic stenosis, including reduced vascular bed and shunt opening.^21^ These findings align with those of Dietrich et al., who observed no basal‐state skin oxygenation changes after TAVI using hyperspectral imaging, a method that does not assess microcirculatory functional reserve, as we did in our study.^22^

The improvement in CI reported in previous studies was observed only in a subset of patients within our cohort.^11^, ^23^, ^24^ A subgroup, exclusively women, exhibited marked CI increases (Figure S4), preserved baseline perfusion (low central‐peripheral T grad), high peripheral shunting (negative arm‐finger T grad), impaired reactive hyperemia (lower PI ratio, reduced StO_2_ peak, and delayed time to peak PI) and elevated baseline VEGF levels, but no postoperative VEGF rise or reduction in large‐artery stiffness, suggesting preserved capillary recruitment and collateral flow despite macrovascular stiffness. The mobilization of functional microcirculatory reserve and endothelial activation could explain these results.

In contrast, the remaining patients represent a more advanced disease phenotype (e.g., higher valvular calcification scores) and overwhelmed adaptive mechanisms as reflected by reduced tissue perfusion (high central‐peripheral T grad). In these patients, the postoperative rise in VEGF, coupled with reductions in large‐artery stiffness and central‐peripheral T grad, suggests a greater vascular remodelling capacity in response to the acute hemodynamic changes induced by TAVI. Supporting this interpretation, we observed that changes in CI were strongly and inversely correlated with changes in plasma VEGF levels at D + 3. We hypothesize that in cases where the vascular tree is markedly reduced due to the adaptation to advanced AS (stroke volume is constrained), a pro‐angiogenic signal is triggered by an increase in flow to promote new vessel formation. Conversely, as observed in women, when the vascular tree remains preserved and a functional microcirculatory reserve can be mobilized, VEGF levels do not rise, as there is no physiological demand for neovascularization.

VEGF levels and dynamic changes correlated with both microvascular and tissue perfusion parameters (Figure S6), underscoring the role of endothelial activation in modulating microvascular function and tissue perfusion under acute hemodynamic stress. Postoperatively, VEGF increased with wide interindividual variability; higher baseline levels and larger rises were associated with postoperative organ dysfunction. This highlights that patients who experienced postoperative organ dysfunction display less downstream vascular reserve as they are forced to stimulate more capillary network (activating the VEGF pathway). An integrated vascular phenotype, impaired compliance and microvascular reserve may limit adaptation to the ‘dam rupture’ of valve opening, thereby increasing the risk for organ dysfunction. These findings are consistent with correlations reported in the literature between microcirculatory parameters and outcomes (fluid balance and diuresis), highlighting the potential value of combined macro‐ and microcirculatory profiling in preoperative risk stratification.^22^ Mechanistically, patients with AS have lower levels of wall shear stress and higher serum VEGF compared to patients without AS.^2^, ^25^ Prior studies have shown increased wall shear stress and VEGF dynamics in AS and after TAVI. Indeed, Horn et al. reported increased flow‐mediated vasodilation, and Ben‐Shoshan et al. observed sustained VEGF elevation at D + 2 and D + 30.^13^, ^26^ Endothelial cells respond to mechanical stress via mechano‐signalling, influencing structure and function; VEGF dynamics were related to both vascular characteristics and reactive hyperemia, further supporting a physiologically plausible link between vascular remodelling, endothelial microvascular response and impaired autoregulation, which predispose to organ dysfunction.^27^ Patients with altered baseline endothelial vasodilatory response are known to experience more organ dysfunction after cardiac surgery.^8^, ^9^ Similar associations exist for other growth factors, such as growth differentiation factor 15 in cardiac surgery and TAVI.^28^, ^29^ Endothelial growth factors are an interesting field of research for future perioperative risk markers.

This exploratory study has notable strengths, including its prospective design, comprehensive multimodal assessment of macro‐ and microcirculatory changes after TAVI, and use of advanced techniques such as NIRS, photoplethysmography, arterial tonometry, intra‐aortic pressure wave analysis and VEGF quantification. Standardized timepoints allowed dynamic perioperative profiling, while intraindividual comparisons reduced variability and strengthened internal validity. Additionally, all assessments were performed using standardized protocols by trained investigators, and the inclusion of objective, quantifiable biomarkers minimized observer bias. Exploration of associations between physiological markers and outcomes provides early insight for future risk stratification.

Limitations include its single‐center pilot design, small sample size and strict inclusion criteria, which limit generalizability. These restrictive conditions were essential for a physiological study. They may not fully reflect the broader real‐world TAVI population, particularly patients requiring alternative access routes or with more complex comorbidities. The relatively short follow‐up period limits the ability to assess long‐term adaptations that may occur beyond the early post‐TAVI phase. Technical issues, early discharges, and incomplete follow‐up affected data completeness. The study's technical requirements for advanced vascular and microcirculatory monitoring, including specialized equipment and offline analyses, may also limit immediate applicability in routine clinical practice. In addition, because each patient served as their control, time‐dependent effects or confounding clinical events (e.g., fluid shifts, sedation effects, medication adjustments) may have influenced physiological parameters. Finally, as an exploratory study with multiple comparisons and no adjustment for multiplicity, there is a risk of type I error and some significant findings may reflect chance associations. Nevertheless, the use of standardized measurement protocols and the consistency of physiological trends across participants enhance internal validity and suggest that similar vascular adaptation patterns may be observed in other centers with comparable expertise.

## 7 CONCLUSION

The present pilot study demonstrates that TAVI rapidly alters macro‐ and microcirculation, reflecting complex vascular adaptations to the relief of chronic ventricular obstruction. TAVI produces a sudden lift in obstruction caused by aortic stenosis with an increase in the vascular strain on the vascular tree adapted to years of severe AS. We observed early significant post‐TAVI improvements in tissue perfusion at D + 1, not correlated with macrocirculatory parameters, despite an initial transient microcirculatory reserve impairment. Baseline endothelial and microvascular status influenced postoperative responses in vascular remodelling capacity and organ function. These findings are hypothesis‐generating and highlight the value of vascular profiling for risk stratification in TAVI, warranting confirmation in larger studies and exploration of whether gender impacts hemodynamic post‐TAVI.

## AUTHOR CONTRIBUTIONS

**Stanislas Abrard**, **Stéphane Noble** and **Karim Bendjelid**: Conceptualization and project administration. **Stanislas Abrard**, **Dyonisios Adamopoulos**, **Georgios Rovas** and **Nikolaos Stergiopulos**: Data curation. **Stanislas Abrard**, **Georgios Rovas** and **Nikolaos Stergiopulos**: Formal analysis. **Karim Bendjelid**: Funding acquisition. **Stanislas Abrard**, **Sarah Mauler**, **Andres Hagerman**, **Raoul Schorer**, **Bernardo Bollen Pinto**, **Christoph Ellenberger** and **Stéphane Noble**: Investigation. **Stanislas Abrard**, **Ivo Neto Silva**, **Dyonisios Adamopoulos**, **Stéphane Noble** and **Karim Bendjelid**: Methodology. **Stanislas Abrard**, **Sarah Mauler**, **Ivo Neto Silva**, **Dyonisios Adamopoulos**, **Andres Hagerman**, **Raoul Schorer**, **Bernardo Bollen Pinto**, **Christoph Ellenberger**, **Stéphane Noble** and **Karim Bendjelid**: Resources. **Dyonisios Adamopoulos**, **Georgios Rovas** and **Nikolaos Stergiopulos**: Software. **Stéphane Noble** and **Karim Bendjelid**: Supervision. **Dyonisios Adamopoulos**, **Stephane Bar**, **Georgios Rovas**, **Nikolaos Stergiopulos**, **Stéphane Noble** and **Karim Bendjelid**: Validation. **Stanislas Abrard**: Visualization and writing—original draft. **Stanislas Abrard**, **Sarah Mauler**, **Ivo Neto Silva**, **Dyonisios Adamopoulos**, **Stephane Bar**, **Andres Hagerman**, **Raoul Schorer**, **Bernardo Bollen Pinto**, **Georgios Rovas**, **Stéphane Noble** and **Karim Bendjelid**: Writing—review and editing.

## FUNDING INFORMATION

This work was supported by mobility grants awarded by Hospices Civils de Lyon (Lyon, France) and departmental funds from the Adult Intensive Care Division, Hôpitaux Universitaires de Genève (Geneva, Switzerland). The funding body had no role in writing the manuscript, nor in the execution of the study, the collection, management, analysis or interpretation of data, or the decision to submit the report for publication.

## CONFLICT OF INTEREST STATEMENT

Stanislas Abrard has received honoraria for consulting from Viatris Inc. (France) and from Public Health Expertise (France). The other authors declare no competing interests.

## CLINICAL TRIAL REGISTRATION

ClinicalTrials.gov NCT 06154642.

## Supporting information


Data S1.

## Data Availability

The datasets used and analyzed during the current study are available from the corresponding author on reasonable request.
