# Supplementary material for: Hemodynamic and microcirculatory early adaptations following transcatheter aortic valve implantation (TAVI): A physiological pilot study
Source: Eur J Clin Invest. 2025 Dec 2;56(1):e70156. doi: 10.1111/eci.70156 (PMC12825404; doi:10.1111/eci.70156)
Supplement: Supplementary file 1 — Data S1. [file ECI-56-e70156-s001.docx]

**Supplement material.**

**Supplement material - Detailed protocol**

*Study design*

This is a prospective, single-center, investigator-initiated, observational, physiological, pilot study conducted at Geneva University Hospitals (HUG).

*Setting*

The study was conducted at HUG, a tertiary academic medical center. Patients were recruited during the preoperative consultation before TAVI, during the inclusion period starting in January 2024 and ending in June 2024. Patients undergo baseline evaluations before TAVI. The exposure was defined as the TAVI procedure performed via the transfemoral route under conscious sedation. Patients were followed for clinical outcomes up to 30 days post-procedure, with data collected during hospitalization and at a dedicated outpatient follow-up visit on day 30.

This study was approved by the Cantonal Ethics Committee of Geneva (Commission Cantonale d'Éthique de la Recherche de Genève) (Chairman Dr. Olivier Hubert) under protocol number 2023-01118 (25^th^ oct. 2023). The study has been conducted following the Declaration of Helsinki and applicable Swiss legal and regulatory requirements. Written informed consent was obtained from all participants before inclusion. Given the observational and non-invasive nature of the microcirculatory assessments, the study was classified as Category A under the Swiss Human Research Ordinance (HRO, Article 7). The study protocol was registered in ClinicalTrials.gov (NCT 06154642) before the inclusion of the first patient in the study. Data were collected and managed following local data protection regulations.

*Participants*

Participant selection was performed by a study investigator based on clinical records and eligibility assessment during the routine preoperative consultation. Eligible participants were adult patients (≥18 years) with severe AS scheduled for transfemoral TAVI at HUG. AS classification was performed following the diagnostic criteria proposed by the European Society of Cardiology Guidelines for the management of valvular disease [1]. Exclusion criteria included non-femoral access for TAVI, concomitant severe valvular disease, planned general anesthesia, pre-existing physical disability for a 6-minute walk test, left ventricular ejection fraction <40%, end-stage renal disease, chronic respiratory failure requiring home oxygen therapy, recent cardiovascular events or vascular procedures (<3 months), active cancer or anti-angiogenic therapy, known cognitive impairment, pregnancy or breastfeeding, and individuals under legal protection or incarceration. Written informed consent is obtained after a reflection period, the day before the TAVI procedure.

Follow-up included five standardized assessment timepoints: baseline (at cath-lab before sedation on the day of TAVI), intraoperative (continuously or before/after implantation for discontinuous measurements), and at 3 hours (H+3), day 1 (D+1), and day 3 (D+3) post-procedure (**Figure 1**). A clinical evaluation was conducted at day 7 (D+7) to assess organ dysfunction and functional capacity. A final follow-up at day 30 documented adverse events, survival, and functional outcomes.

*Variables*

The primary outcome was the change in microcirculatory parameters after TAVI, assessed at predefined timepoints including baseline, H+3, D+1, and D+3. These parameters included arterial stiffness measured using tonometry, the cutaneous temperature gradient (T°grd) measured as the difference between the forearm and fingertip, the reactive hyperemia assessed by tissular near-infrared spectroscopy (NIRS) and photoplethysmography, and plasma VEGF concentration.

Secondary outcomes included the occurrence of early postoperative organ dysfunction within 7 days defined as a composite of acute kidney injury (AKI) according to KDIGO stage one or higher [2], cardiovascular failure requiring vasopressor or inotropic support for more than 2 hours and postoperative neurocognitive disorder assessed using the 3D Confusion Assessment Method (3D-CAM)[3] on D+1. Additional outcomes included functional capacity was assessed by a six-minute walk test performed on day six, with results expressed in absolute distance and percentage of predicted value, the occurrence of major adverse cardiovascular events (MACEs) within 30 days such as myocardial infarction, unstable angina, stroke or transient ischemic attack, cardiovascular death or elevated high sensitivity troponin T greater than or equal to ≥ 65 nanograms per liter [4] and major adverse kidney events (MAKEs) defined as death, dialysis dependence or a decrease in estimated glomerular filtration rate > 25 % from baseline. All-cause mortality within 30 days following the procedure is also recorded.

Predictors and potential confounders included comorbid conditions such as diabetes, chronic kidney disease and hypertension taking into account clinical risk scores including EuroSCORE II [5], STS [6], STS TAVR [7], echocardiographic indices such as AS grade and stage [8], and biological markers including baseline VEGF, brain natriuretic peptide (NTproBNP), creatinine and troponin levels.

*Data sources, measurements*

All variables were prospectively collected using standardized procedures from patients’ clinical examinations and medical records and recorded in a secure electronic case report form. Figure 1 shows the chronology of the measurements. Microcirculatory parameters were measured on resting participants instructed not to move or speak during the procedure. Arterial stiffness of large and small vessels was measured noninvasively using applanation tonometry with a pressure sensor placed on the radial artery to assess pulse wave characteristics (HDI Pulsewave CR-2000 research cardiovascular profiling system, Eagan, MN, USA). The T°grd was measured using surface thermometers (Biopac systems Inc. CA, USA) placed on the forearm, midway between the wrist and the elbow, and the fingertip opposite to the nailbed, with the gradient calculated as the difference in skin temperature between the two sites. Reactive hyperemia was assessed via a vaso-occlusion test (VOT) using a pneumatic cuff inflated (50 mmHg up arterial pressure) for three minutes followed by measurement of reperfusion using photoplethysmography on the index finger (Masimo Radical 7, Masimo Corp, CA, USA) and tissue oxygen saturation by NIRS (INVOS 5100C, Medtronic, CO, USA). Parameters collected include perfusion index peak amplitude, time to peak from photoplethysmography, as well as tissue resaturation rate from NIRS. Plasma VEGF concentrations were measured from venous blood samples taken via existing vascular access using ELISA methodology (R&D Systems Inc. MN, USA) and analyzed in the hospital laboratory. The intra-aortic pressure signal was recorded using standard cath-lab monitoring systems (Biopac systems Inc. CA, USA) and stored for offline analysis as described by Pagoulatou et al. [9]

The complete transthoracic echocardiography (TTE) in supine position was performed before and between D+1 and D+2 to the TAVI in all study participants by an experienced cardiologist. Data on left ventricular geometry and proximal velocity profile acquired in the left ventricular outflow tract via Pulsed Wave Doppler in the standard apical 5-chamber view were collected. The aortic flow waveform was subsequently derived after calibration for the invasively measured systolic volume. Aortic valve assessment and qualitative evaluation of other valve abnormalities (mitral, tricuspid) were extracted from the standard echocardiographic reports.

Clinical outcomes were assessed through review of electronic medical records, physical examination, structured interviews, and standardized tests. Laboratory values, including NTproBNP, creatinine, and other clinical biomarkers, were obtained from routine blood draws performed in the hospital laboratory following standard protocols. Functional capacity was evaluated using the six-minute walk test performed by trained staff with results recorded in meters and expressed as a percentage of predicted values adjusted for age, sex, and body size [10].

*Bias*

All consecutive patients undergoing transfemoral TAVI who met eligibility criteria were screened and invited to participate during routine preoperative consultation, minimizing subjective patient selection. Assessments were performed using standardized procedures at all predefined time points. Data interpretation was conducted offline, and investigators analyzing outcomes were blinded to clinical events. Potential confounding variables were included as covariates in the exploratory models.

Since each patient served as their control for primary outcome comparisons, intraindividual variability was controlled, which strengthens the internal validity of the physiological assessments. As this is a single-center pilot study with no control group, external validity is limited, but internal consistency is reinforced through uniform protocols and follow-up procedures across all participants. Briefly, all TAVI procedures were performed under conscious sedation following the institutional protocol at HUG. Sedation was induced using titrated doses of dexmedetomidine administered via continuous infusion. The level of sedation was monitored clinically and adjusted to maintain patient comfort. Supplemental oxygen was administered via face mask as required. A dedicated anesthesiology team was present throughout the procedure to ensure hemodynamic stability and provide sedation management. Intraoperative hypotension was managed according to institutional standards. Initial treatment included ephedrine bolus administration as a first-line agent in cases of mild hypotension associated with low heart rate, followed by neosynephrine for hypotension with preserved or elevated heart rate. For persistent hypotension or signs of hemodynamic instability, norepinephrine was initiated via peripheral venous access. In cases of patient agitation or discomfort unresponsive to titrated sedation, conversion to general anesthesia was considered. General anesthesia was induced using standard agents such as propofol and fentanyl, with airway management using a laryngeal mask.

Standard intraoperative monitoring included intra-arterial access was established via the left radial artery and the bilateral femoral arteries for catheterization, and also enables acquisition of the aortic pulse pressure waveform in real time.

*Statistical methods*

Categorical variables were reported as counts with percentages, and continuous variables as medians with interquartile ranges. Primary analyses compared within-patient quantitative changes using the Wilcoxon signed-rank test. Secondary analyses compared patients grouped by postoperative organ dysfunction or adverse outcomes, employing the Mann-Whitney U test for continuous and Fisher's exact test for categorical variables.

No formal sensitivity analyses or adjustments for multiple comparisons were planned due to the study's exploratory nature and small sample size.

The planned sample size of this physiological study of 20 patients was determined according to our institution’s annual TAVI procedure volume (80–90 cases), ensuring the feasibility of recruitment within a 12-month period. Analyses were conducted with SPSS software version 23 (IBM Corp., USA). Statistical significance was defined as a two-sided p-value <0.05. Missing data were imputed only in linear models; otherwise, analyses used available data.

**References**

1. Vahanian A, Beyersdorf F, Praz F, Milojevic M, Baldus S, Bauersachs J, et al. 2021 ESC/EACTS Guidelines for the management of valvular heart disease. European Heart Journal. 2022 Feb 12;43(7):561–632.

2. Khwaja A. KDIGO Clinical Practice Guidelines for Acute Kidney Injury. Nephron Clin Pract. 2012 Aug 7;120(4):c179–84.

3. Marcantonio ER, Ngo LH, O’Connor M, Jones RN, Crane PK, Metzger ED, et al. 3D-CAM: Derivation and Validation of a 3-Minute Diagnostic Interview for CAM-Defined Delirium: A Cross-sectional Diagnostic Test Study. Ann Intern Med. 2014 Oct 21;161(8):554.

4. Writing Committee for the VISION Study Investigators, Devereaux PJ, Biccard BM, Sigamani A, Xavier D, Chan MTV, et al. Association of Postoperative High-Sensitivity Troponin Levels With Myocardial Injury and 30-Day Mortality Among Patients Undergoing Noncardiac Surgery. JAMA. 2017 Apr 25;317(16):1642.

5. Nashef SA, Roques F, Sharples LD, Nilsson J, Smith C, Goldstone AR, et al. EuroSCORE II. Eur J Cardiothorac Surg. 2012 Apr;41(4):734–44; discussion 744-5.

6. O’Brien SM, Feng L, He X, Xian Y, Jacobs JP, Badhwar V, et al. The Society of Thoracic Surgeons 2018 Adult Cardiac Surgery Risk Models: Part 2—Statistical Methods and Results. The Annals of Thoracic Surgery. 2018 May;105(5):1419–28.

7. Figulla HR, Lauten A, Hamm C, Lange R, Sack S, Mohr FW. TCT-715 Developing of a TAVR risk score for the prediction of mortality after transcatheter aortic valve replacement. Journal of the American College of Cardiology. 2014 Sep;64(11):B210.

8. Généreux P, Pibarot P, Redfors B, Mack MJ, Makkar RR, Jaber WA, et al. Staging classification of aortic stenosis based on the extent of cardiac damage. European Heart Journal. 2017 Dec 1;38(45):3351–8.

9. Pagoulatou S, Stergiopulos N, Bikia V, Rovas G, Licker MJ, Müller H, et al. Acute effects of transcatheter aortic valve replacement on the ventricular-aortic interaction. American Journal of Physiology-Heart and Circulatory Physiology. 2020 Dec 1;319(6):H1451–8.

10. Enright PL, Sherrill DL. Reference Equations for the Six-Minute Walk in Healthy Adults. Am J Respir Crit Care Med. 1998 Nov 1;158(5):1384–7.


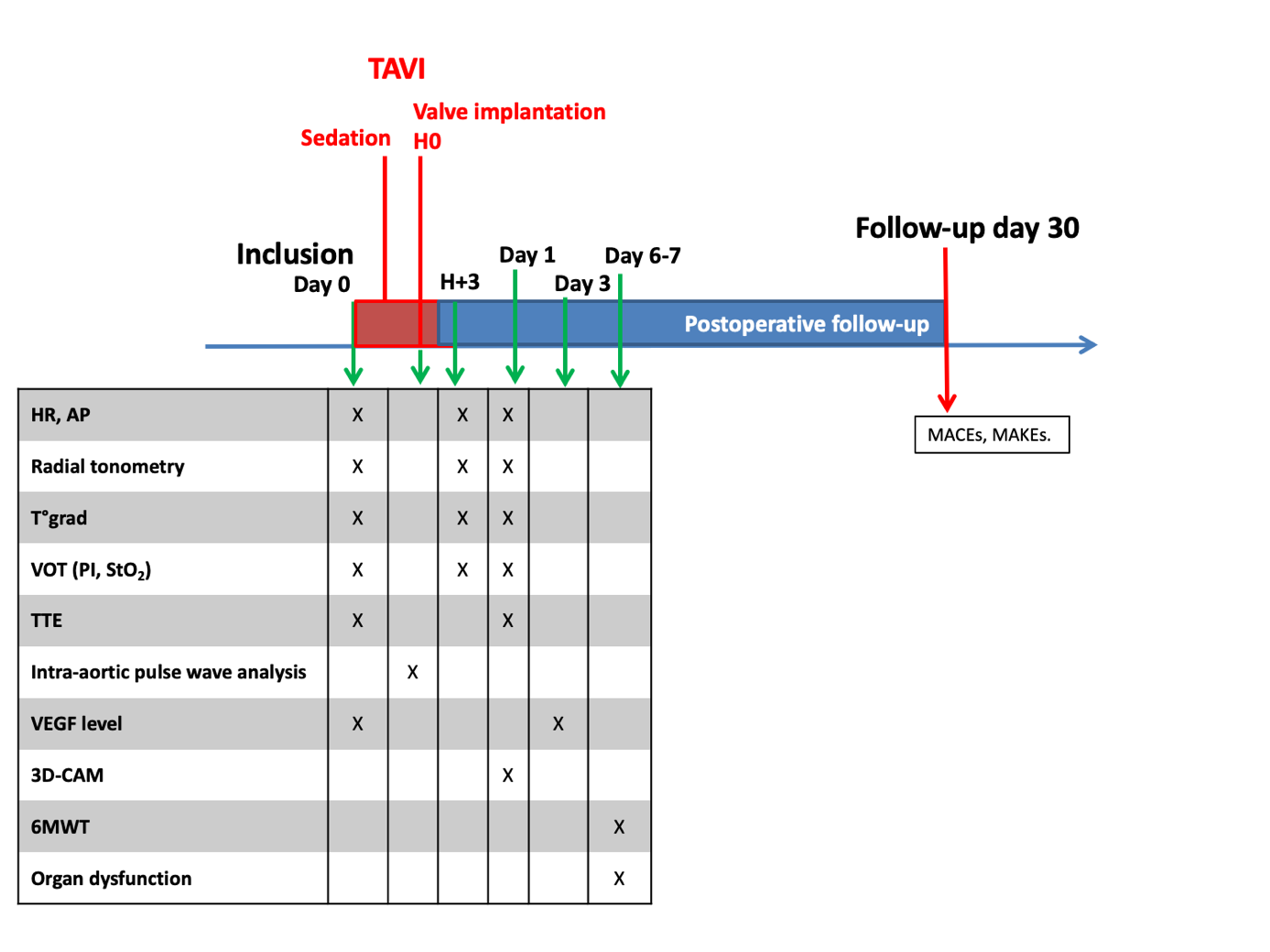


**Supplement material - Figure 1: Study follow-up.** HR: heart rate; AP: arterial pressure; T°grad: temperature gradient; VOT: vaso occlusion test; PI: perfusion index; StO_2_: tissue oxygen saturation; TTE: transthoracic echocardiography; VEGF: vascular endothelium growth factor; 3D-CAM: 3D confusion assessment method; 6MWT: six-minute walk test; MACEs: major adverse cardiovascular events; MAKEs: major adverse kidney events.

**Supplement material - Figure 2: Evolution of reactive hyperemia after vaso-occlusion test between inclusion and day+1 after TAVI.**

**
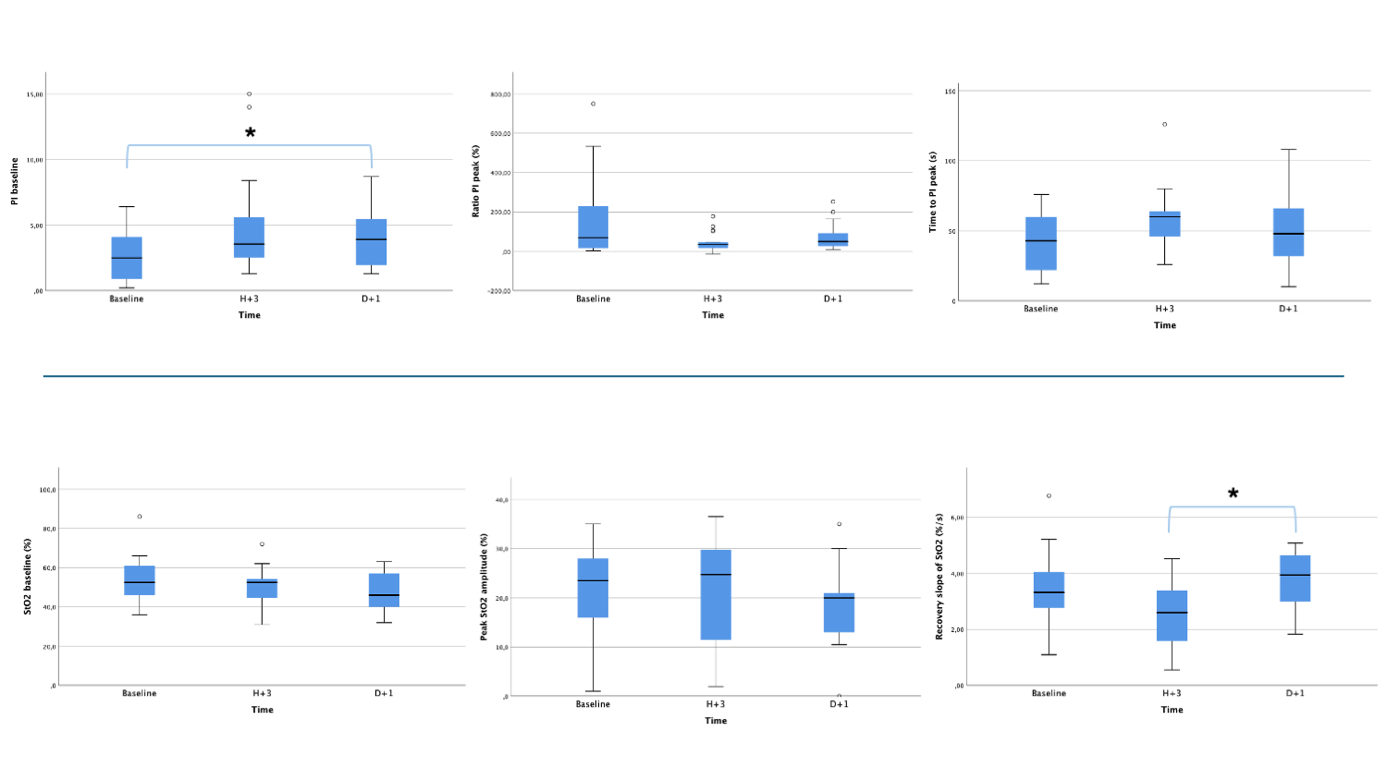
**

Perfusion index (PI) baseline (before vaso-occlusion test), PI ratio of peak amplitude after vaso-occlusive test on baseline value, time to reach the peak of PI after a vaso-occlusive test, tissular oxygen saturation (StO_2_) baseline (before vaso-occlusion test), peak amplitude of StO_2_ after vaso-occlusive test, time to reach the peak of StO_2_ after a vaso-occlusive test are presented at 3 different timepoints. Measurements were compared by the Wilcoxon test. * p < 0.05

**Supplement material - Figure 3: Evolution of Vascular Endothelium Growth Factor.**


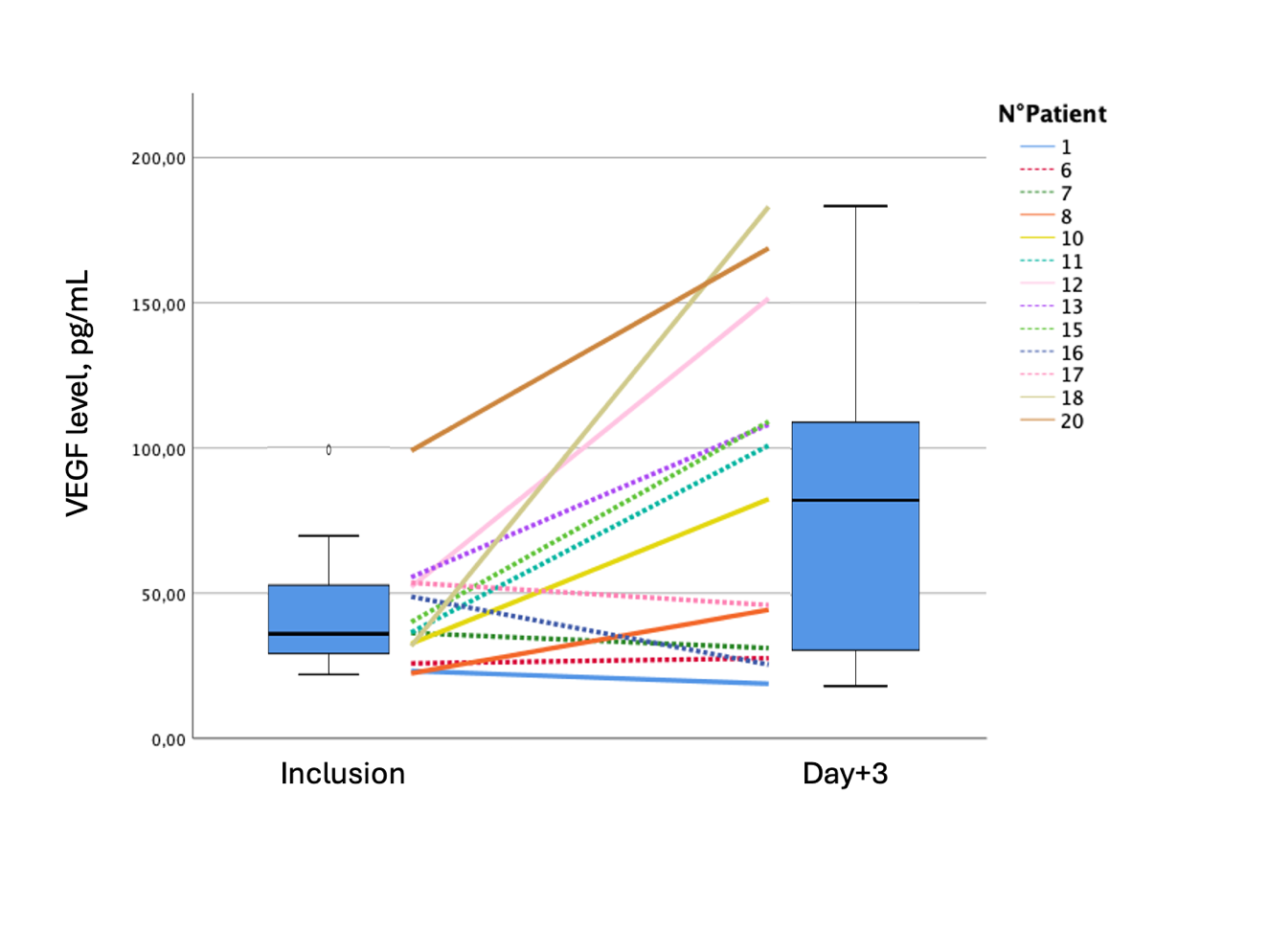


Pre and day+3 VEGF levels were compared by the Wilcoxon test: *p* = 0.033. Female patients are shown with dotted lines and male patients with solid lines.

**Supplement material - Figure 4: Individual intraoperative evolution of cardiac index before and after TAVI.**

**
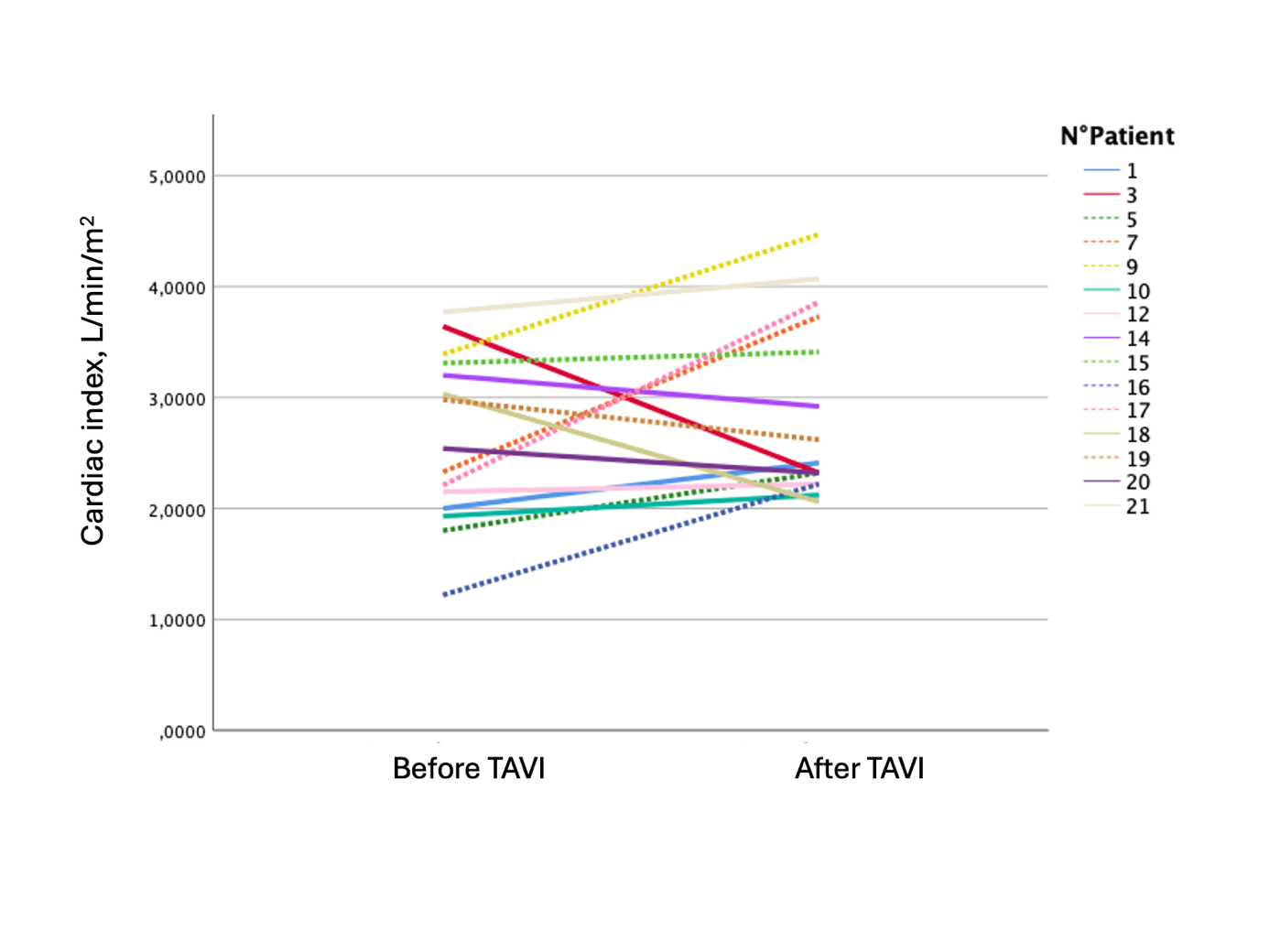
**

Cardiac index obtained by analyzing the intraoperative intra-aortic pressure signal before and after TAVI. Female patients are shown with dotted lines and male patients with solid lines.

**Supplement material - Figure 5: Matrix of correlation between evolution of macrovascular parameters.**

**
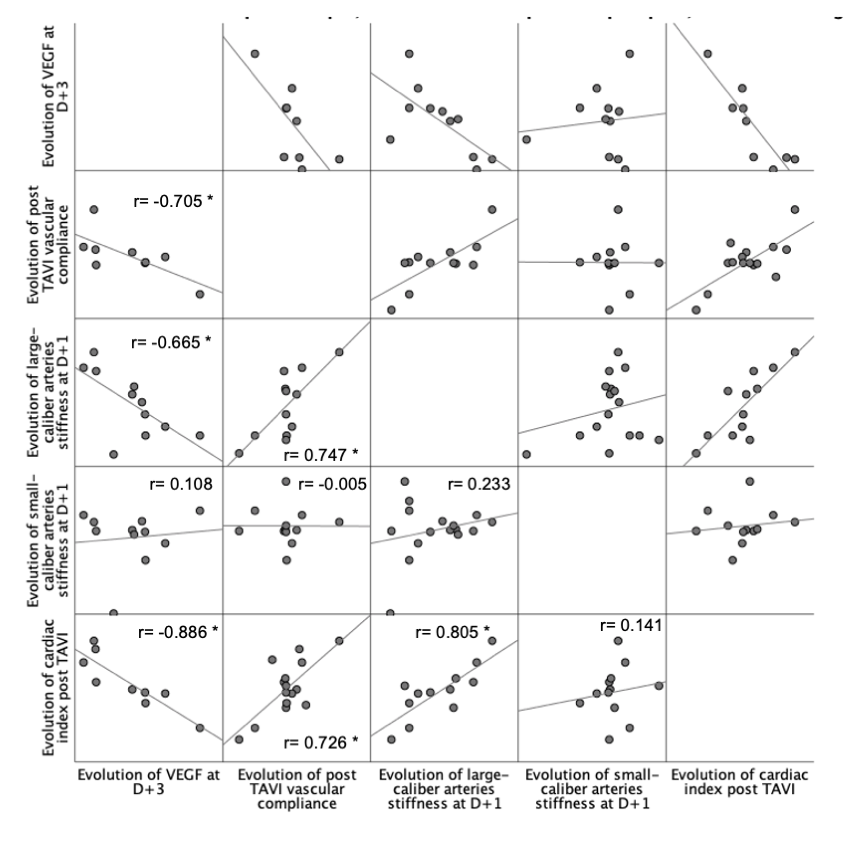
**

Correlations were evaluated by Pearson correlation index. * p < 0.05

**Supplement material - Figure 6: Matrix of correlation between VEGF and evolution of microvascular parameters.**

**
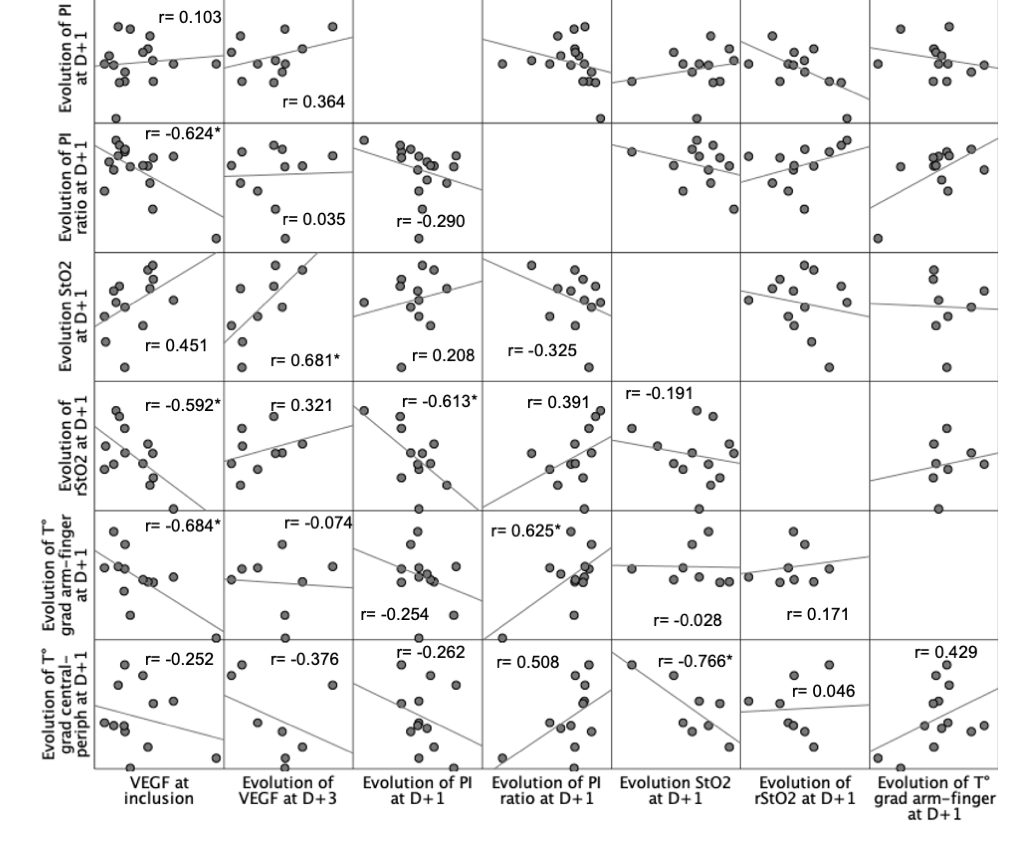
**

Correlations were evaluated by Pearson correlation index. * p < 0.05

**Supplement material - Figure 7: Matrix of correlation between evolution of micro- and macro-vascular parameters.**

**
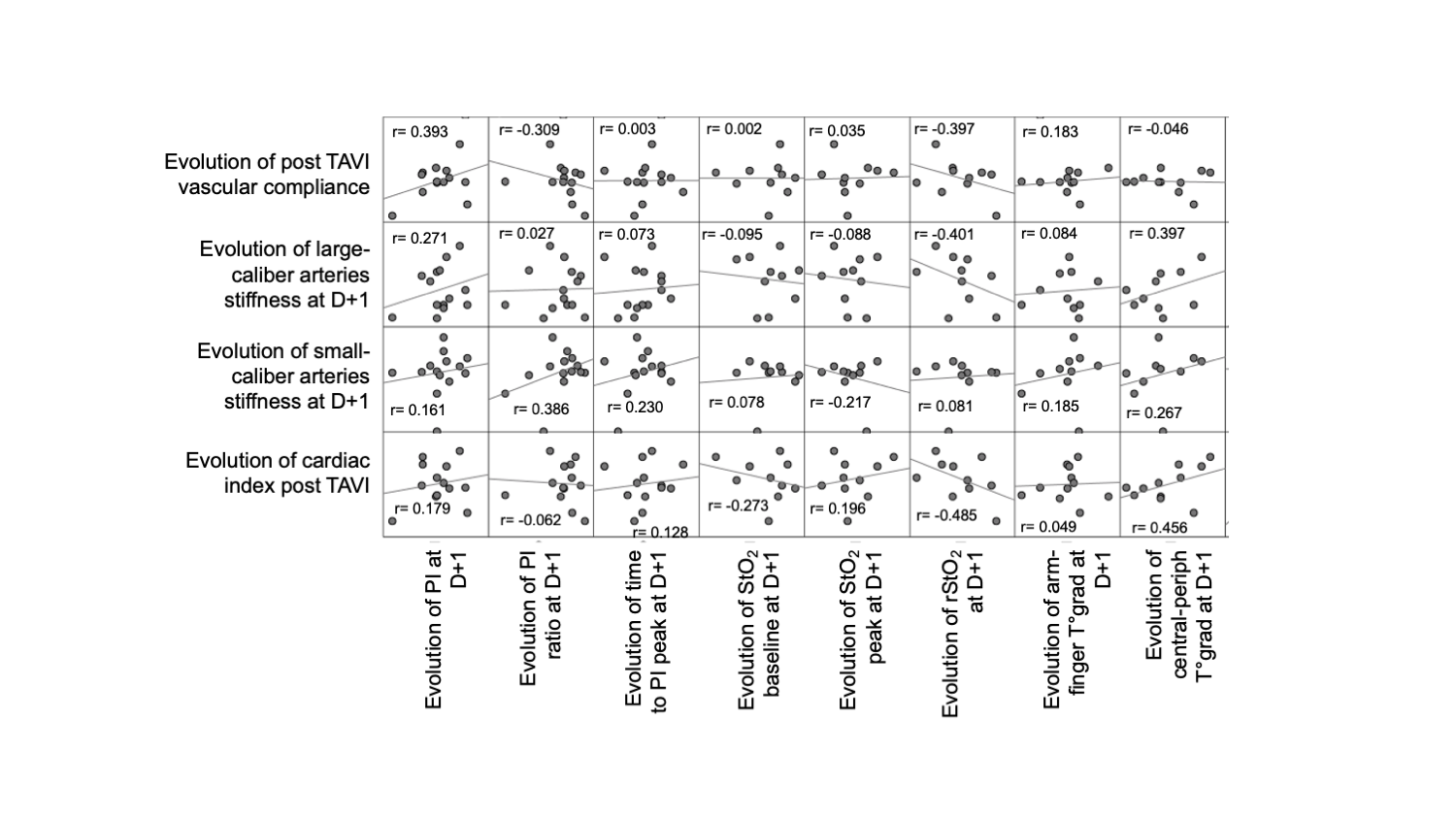
**

Correlations were evaluated by Pearson correlation index. No correlation reach the statistical significance level (p < 0.05).

**Supplement material - Figure 8: Dispersion of cardiac index evolution by VEGF evolution.**


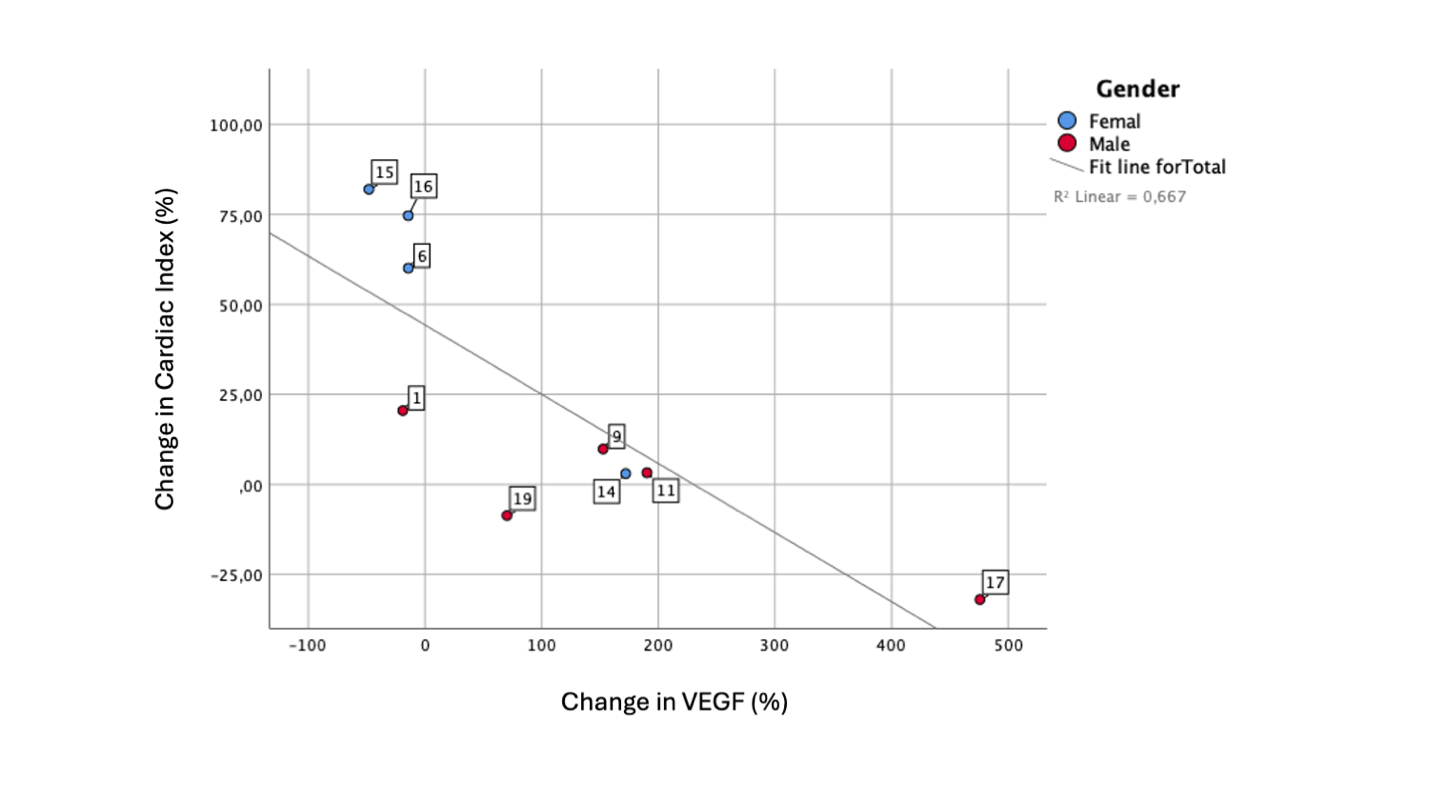


The change in cardiac index before/after TAVI and the change in VEGF before/on day 3 are presented as a percentage of their initial value. Each point is designated by the patient's number and gender (blue = female; red = male). The correlation is measured by Pearson's coefficient: r = -0.806; *p* = 0.009.

**Supplement material - Table 1: Comparison of microcirculatory and tissue perfusion changes after TAVI in patients with or without postoperative organ dysfunction.**

| Parameters | No organ dysfunction | Organ dysfunction | p-value |
| --- | --- | --- | --- |
| Evolution of PI baseline at D+1 | 1.0 [-1.2-1.9] | 1.4 [0.4-3.5] | 0.301 |
| Evolution of Ratio PI peak at D+1, % | -39 [-137-88] | -58 [-205-29] | 0.660 |
| Evolution of Time to PI peak at D+1, s | -2 [-37-9] | 18 [-4-32] | 0.062 |
| Evolution of StO_2_ baseline at D+1, % | -7 [-13, -2] | 0 [-2.5-4] | 0.008 |
| Evolution of Peak StO_2_ amplitude at D+1, % | 1 [-3-6] | -7 [-11, -4] | 0.008 |
| Evolution of rStO_2_ at D+1, %/s | 0.4 [-0.1-1.7] | 0.5 [-0.9-0.9] | 0.445 |
| Evolution of Arm-finger T°grad at D+1, °C | 3.3 [1.6-5.3] | 2.2 [-0.7-3.4] | 0.435 |
| Evolution of Central-periph T°grad at D+1, °C | -3.2 [-3.5-2.2] | -4.0 [-6.6, -1.0] | 0.127 |

PI: perfusion index; rStO_2_: recovery slope of tissular oxygen saturation; rStO_2_: tissular oxygen saturation, T°grad: temperature gradient. Measurements were compared by the Mann-Whitney test.

**Supplement material - Table 2: Comparison of patient characteristics and evolution between terciles of cardiac index evolution after TAVI.**

| Characteristic | Evolution CI< 0.5 | Evolution CI> 0.5 |
| --- | --- | --- |
| N (%) | 10 (67) | 5 (33) |
| Male sex * | 8 (53) | 0 * |
| Age at the enrollment, years | 81 [76-87] | 84 [82-90] |
| Body mass index, kg/m^2^ | 23.9 [22.0-25.3] | 26.0 [24.3-30.6] |
| Medical conditions  Diabetes mellitus  Hypertension  Chronic kidney disease | 2 (13)  8 (53)  3 (20) | 2 (13)  4 (27)  5 (33) |
| Cardiopathy  Aortic stenosis  Gradient (mmHg)  Valve calcic score | 55 [46-60]  4043 [2711-5077] | 48 [31-73]  1820 [1448-3717] |
| EuroSCORE II  NTproBNP, pg/mL  Baseline VEGF, pg/mL | 6.5 [4.0-8.3]  1176 [322-3839]  32.6 [29.5-46.2] | 7.0 [6.0-9.0]  1230 [88-2961]  53.6 [42.6-62.8] |
| Preoperative hemodynamic:  Systolic arterial pressure, mmHg  Mean arterial pressure, mmHg  Diastolic arterial pressure, mmHg  Pulse rate, bpm | 135 [113-155]  95 [86-110]  72 [63-87]  69 [56-85] | 150 [140-161]  104 [94-117]  72 [53-85]  83 [75-91] |
| Preoperative microcirculation:  Arm-finger T°grad, °C *  Central-peripheral T°grad, °C *  PI baseline  Ratio PI peak, %  Time to PI peak, s  StO2 baseline, %  Peak StO2 amplitude, %  rStO2, %/s | 0.64 [0.27-3.25]  6.78 [4.05-8.99]  1.70 [0.95-2.95]  86 [39-315]  23 [19-56]  52 [46-65]  26 [17-32]  3.1 [2.4-3.9] | -2.68 [-3.60, -0.94] *  2.68 [1.12-3.35] *  3.30 [1.85-5.35]  16 [8-142]  60 [39-72]  55 [48-65]  16 [4-26]  3.5 [1.8-5.4] |
| Postoperative evolution:  Postoperative complications, n (%)  Evolution of VEGF at D+3, pg/mL *  Evolution of vascular resistance at D+1, dyne.s.cm-^5^  Evolution of post TAVI vascular compliance, mL/mmHg  Evolution of stiffness of large-caliber arteries at D+1, mL/mmHg *  Evolution of stiffness of small-caliber arteries at D+1, mL/mmHg  Evolution of T°grad arm-finger at D+1, °C  Evolution of T°grad central-periph at D+1, °C * | 6 (40)  69.4 [36.3-112.3]  434 [-202-1347]  -0.16 [-0.30, -0.50]  -4.4 [-5.4,-1.4]  -0.6 [-1.4-0.9]  1.98 [-1.47-3.55]  -3.79 [-6.89,-3.51] | 3 (20)  -7.7 [-23.4, -6.4] *  -107 [-117,-7]  0.04 [-0.27-0.36]  0.9 [-1.0-1.6] *  0.4 [-0.4-0.8]  2.34 [1.99-3.10]  0.34 [-1.17-2.49] * |

The evolution of the cardiac index (CI) was dichotomized between patients with no clinically significant increase in the CI (1st and 2nd tercile: evolution of the CI < 0.5 L/min/m^2^) and patients with a significant evolution of the CI (3rd tercile, evolution > 0.5 L/min/m^2^). Comparison by the Mann-Withney and the Fisher exact test. * p < 0.05

T°grad, temperature gradient. PI, perfusion index. StO2, tissular oxygen saturation. rStO2, slope of tissular resaturation after vaso-occlusion test.
